# Detection of the *MYD88* p.L265P Mutation in the CSF of a Patient With Secondary Central Nervous System Lymphoma

**DOI:** 10.3389/fonc.2018.00382

**Published:** 2018-09-20

**Authors:** Soheil Zorofchian, Guangrong Lu, Jay-Jiguang Zhu, Dzifa Y. Duose, Justin Windham, Yoshua Esquenazi, Leomar Y. Ballester

**Affiliations:** ^1^Department of Pathology and Laboratory Medicine, University of Texas Health Science Center, Houston, TX, United States; ^2^Vivian L. Smith Department of Neurosurgery, University of Texas Health Science Center, Houston, TX, United States; ^3^Department of Translational Molecular Pathology, UT MD Anderson Cancer Center, Houston, TX, United States

**Keywords:** *MYD88*, central nervous system lymphoma, liquid biopsy, cerebrospinal fluid, digital droplet PCR, CSF

## Abstract

Primary Central Nervous System Lymphoma (PCNSL) and Metastatic (or Secondary) Central Nervous System Lymphoma (MCNSL) are rare central nervous system (CNS) malignancies that exhibit aggressive clinical behavior and have a poor prognosis. The majority of CNS lymphomas are histologically classified as diffuse large-B cell lymphoma (DLBCL). DLBCL harbors a high frequency of mutations in *MYD88* and *CD79b*. The *MYD88* p.L265P mutation occurs at high frequency in CNS lymphoma and is extremely rare in non-hematologic malignancies. Currently, brain biopsy is considered the gold standard for CNS lymphoma diagnosis. However, brain biopsy is invasive, carries a risk of complications, and can delay initiation of systemic therapy. Circulating tumor DNA (ctDNA) in the cerebrospinal fluid (CSF) can be utilized to detect tumor-derived mutations. Testing of CSF-ctDNA is a minimally-invasive methodology that can be used to assess the genomic alterations present in CNS malignancies. We present a case of an 82-year-old man with a history of testicular lymphoma who presented with speech difficulty and a multifocal enhancing left inferior frontal mass. Analysis for both CSF-cytology and flow cytometry did not show evidence of neoplastic cells. A brain biopsy was performed and microscopic examination showed DLBCL. We isolated CSF-ctDNA and used droplet digital PCR (ddPCR) to detect the most common lymphoma-associated mutations in *MYD88*, L265P, and V217F. In conjunction, we evaluated the patient-matched CNS lymphoma tissue for *MYD88* mutations. We detected the *MYD88* p.L265P mutation in formalin fixed paraffin embedded (FFPE) tissue from the brain biopsy and the CSF-ctDNA. In contrast, both the tumor tissue and the CSF ctDNA were negative for the *MYD88* p.V217F mutation. This study shows that testing CSF ctDNA for *MYD88* mutations is a potentially minimally-invasive approach to diagnosing patients with suspected CNS lymphomas.

## Introduction

Lymphoma of the central nervous system (CNS) includes two major subtypes: primary central nervous system lymphoma (PCNSL) arisen and confined to the CNS and metastatic or secondary CNS lymphoma (MCNSL), which originates at sites outside the CNS (1, 2). Accounting for 1–5% of all CNS tumors, PCNSL are mainly defined as a rare group of non-Hodgkin lymphomas that are most frequently morphologically classified as diffuse large B-cell lymphoma (DLBCL) (3–5). Magnetic resonance imaging (MRI) is helpful in the diagnosis of CNS lymphoma and often shows single or multiple periventricular, homogeneously enhancing lesions. Nonetheless, due to low specificity, MRI is insufficient for accurate CNS lymphoma diagnosis and a brain biopsy is often performed prior to treatment (6). Cerebrospinal fluid (CSF) cytology and flow cytometry can help to establish a diagnosis of CNS lymphoma. However, both methodologies have a very low sensitivity (7). An accurate diagnosis of CNS lymphoma is important to guide appropriate treatment, which frequently includes high-dose methotrexate, with or without other chemotherapeutic agents, and with or without radiation therapy (8). Surgical removal of CNS lymphoma is not routinely performed as these tumors typically respond to systemic therapies. Therefore, the identification of minimally-invasive tumor biomarkers that facilitate the diagnosis of CNS lymphoma is of great clinical interest.

Studies on the genomic alterations of CNS lymphoma have reported a high frequency of mutations in the myeloid differentiation factor 88 (*MYD88*) gene. Specifically, the p.L265P amino acid substitution is the most commonly occurring *MYD88* mutation in CNS lymphoma (9–11). *MYD88* is an adaptor protein that transduces signals from Toll-like receptors (TLRs) and activates interleukin-1 (IL-1) and IL-18 receptor signaling pathways. The *MYD88* p.L265P mutation is also present in virtually all cases of Waldenstrom macroglobulinemia (12) and at a lower frequency in systemic lymphomas. Here, we describe the case of a patient with a history of testicular lymphoma and secondary CNS involvement in which we successfully detected the *MYD88* p.L265P mutation in CSF using droplet digital PCR (ddPCR) as wells as discuss the potential application of this assay for minimally-invasive diagnosis of CNS lymphomas.

## Background

**Figure 1:**
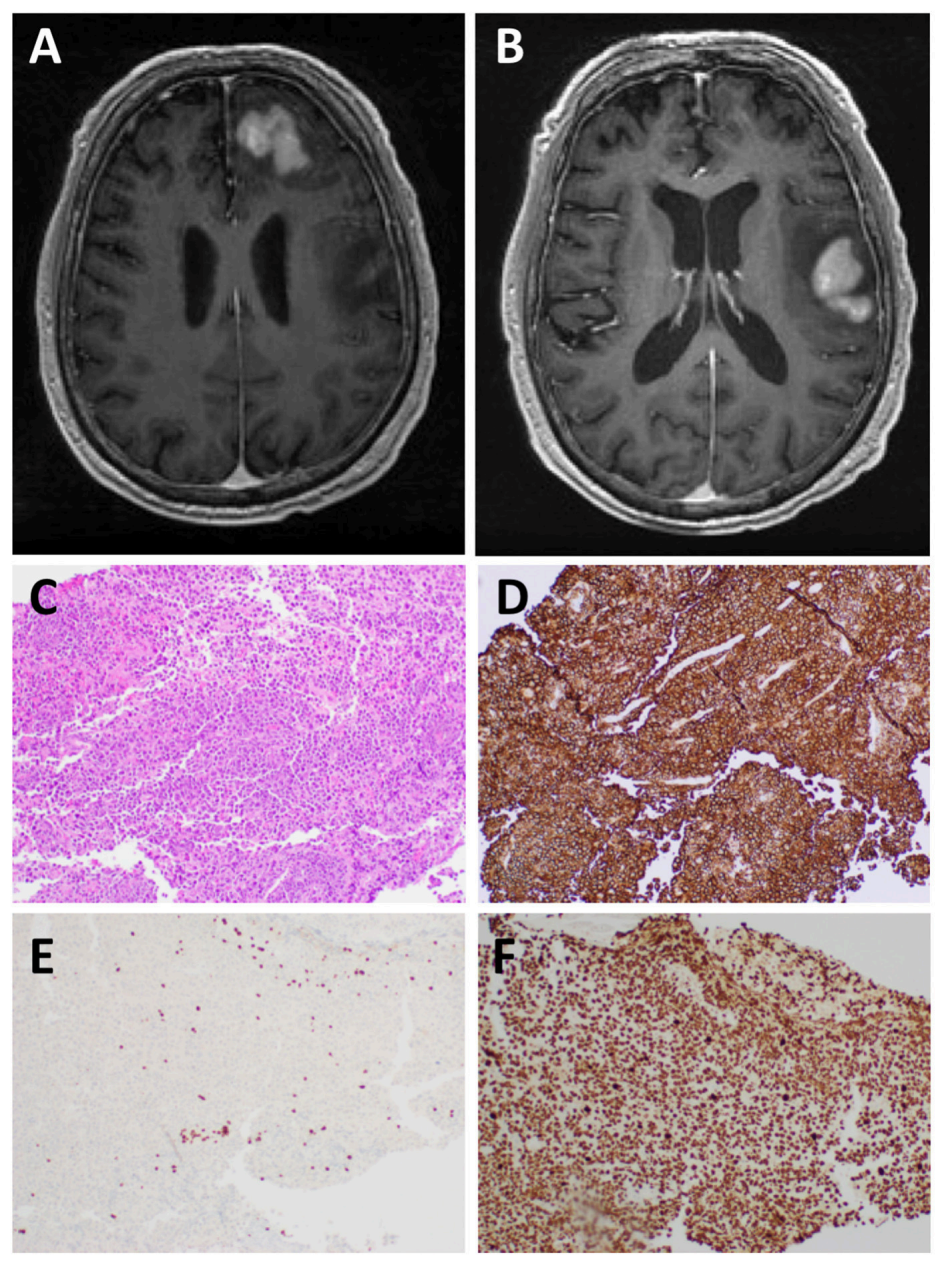
MRI and histological examination. Preoperative MRI of the brain: **(A,B)** There are two T1 hypointense T2 hyperintense mass lesions in the left frontal lobe subcortical white matter and involving the cortex with heterogeneous enhancement measuring 3.2 x 3.1 and 4.5 x 3.3 cm, respectively. There is surrounding vasogenic edema and small areas of restricted diffusion. Microscopic examination of the left frontal lobe biopsy showed diffuse involvement of the brain parenchyma by Diffuse Large B-cell lymphoma: **(C)** H&E staining, **(D)** CD20+ B-cells, **(E)** CD3+ T-cells, and **(F)** Ki67 shows a markedly elevated proliferation index (>95%).

An 82-year-old man, former smoker, presented with a testicular mass on July of 2015. An orchiectomy was performed and microscopic examination of the lesion showed the presence of diffuse large B-Cell lymphoma. Evaluation of the bone marrow at the time did not show evidence of involvement by DLBCL. The patient was treated with chemotherapy, which he tolerated well. In addition, he received radiation therapy. On August of 2015, Pre-operative CSF cytology revealed the presence of atypical lymphocytes insufficient to make a definitive diagnosis of CNS disease. He was admitted to the hospital on February of 2016 due to slurring of his speech. A CT scan of the head was performed and revealed multiple hypodense areas along the frontal and parietal lobes. On MRI, there were two enhancing lesions in the left frontal lobe suggesting the possibility of metastatic CNS lymphoma (Figure 1). Given those findings, the patient underwent treatment with high-dose Methotrexate and Rituxan with significant symptomatic relief noted after the initial 4 cycles. He then reported progressive neurological decline including confusion, falls, seizures, and aphasia; and MRI demonstrated an increase in size of the brain lesions. At this time, the decision was made to perform a brain biopsy for confirmation of diagnosis and symptomatic relief. The patient underwent a brain biopsy which demonstrated secondary brain involvement by DLBCL. Treatment was continued with high-dose Methotrexate and Rituxan, with good tolerance. On follow up, MRI of the spine was performed due to a clinical Brown-Sequard syndrome with a sensory level at T6-T8, right leg hemiplegia, and left sided sensory loss at T6-8 levels. A second CSF cytology examination, performed ~4 weeks after surgery and after treatment with steroids, was negative. The spinal MRI demonstrated abnormal signal and minimal enhancement in the central array of the thoracic cord at the level of T8 suggestive of lymphoma.

## Histologic findings

Microscopic examination of tissue sections from the brain biopsy showed a small tissue fragment with discohesive round cells. The cells had a high nucleus-to-cytoplasm ratio. There was nuclear pleomorphism and numerous cells demonstrated prominent nucleoli. Mitotic figures were readily identified, including atypical forms. In addition, there were abundant apoptotic bodies. Immunostaining showed that the large tumor cells exhibited strong and diffuse membranous staining for CD20 and *CD79b* (Figure 1). There were very few CD3 positive T-cells in the background, all of which were small and mature lymphocytes, in contrast to the very large atypical cells highlighted by the CD20 and CD79a immunostains. The Ki-67 labeling index was >95%. A diagnosis of DLBCL was made.

## CSF-derived circulating tumor DNA (CtDNA) analysis

Formalin-fixed paraffin-embedded (FFPE) tissue was used to extract DNA using QIAamp DNA FFPE Tissue Kit (Qiagen, Valencia, CA, USA). A lumbar puncture was performed (L3-L4) ~4 weeks after surgery and 13 ml of CSF were collected. One milliliter of CSF was used for ctDNA isolation (13). CSF was centrifuged (1,000 g, 10 min, 4°C) and the CSF supernatant was stored at −80°C until the time of analysis. CtDNA was extracted using QIAamp Circulating Nucleic Acid Kit (Qiagen, Hilden, Germany) according to the vendor's instructions. DNA concentration and purity were analyzed using Qubit (Qubit dsDNA HS Assay Kit, Invitrogen, Life Technologies).

**Figure 2:**
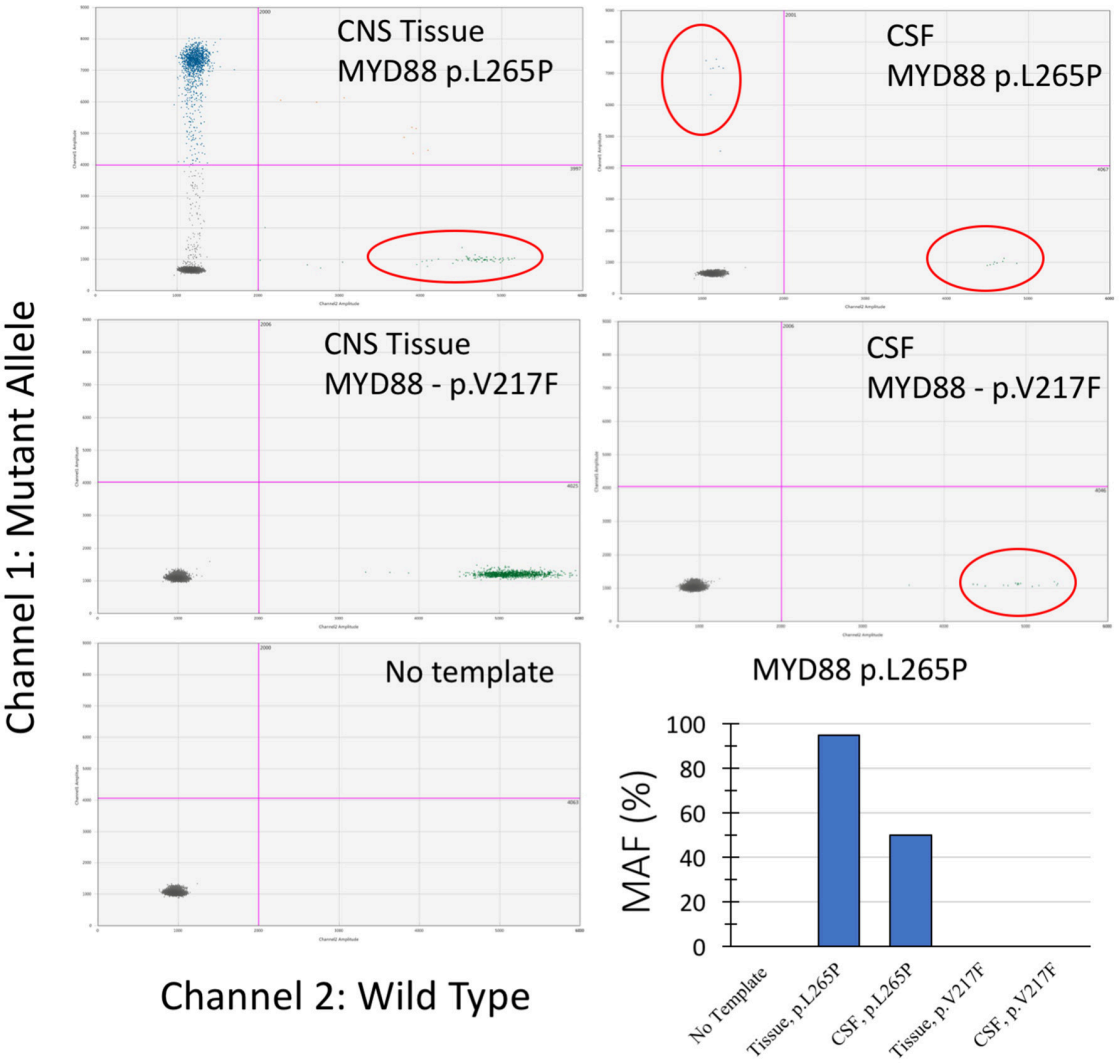
Droplet digital PCR results from examination of the CNS tissue and the CSF-ctDNA for *MYD88* mutations. Quadrant statistics scatter plot (lower left quadrant shows negative cluster; lower right quadrant shows wild-type cluster; upper left quadrant shows mutant cluster; upper right quadrant shows double positive). Both, CNS tissue and CSF-ctDNA showed positive mutant droplets for *MYD88* p.L265P, while no *MYD88* p.V217F mutant droplets were detected. The representative bar chart illustrates the percentage value of mutation allele frequency (MAF) for the CNS tissue and the CSF-ctDNA.

Droplet digital polymerase chain reaction (ddPCR) mutation detection assay for *MYD88* p.L265P and p.V217F mutations were purchased from Bio-Rad (Hercules, CA, USA). The ddPCR mutation detection assay is a 20X concentrated, ready-to-use primer-probe mix optimized for use with ddPCR supermix for probes. The droplet generation process was performed using the Bio-Rad QX-100 emulsification device following the manufacturer's instructions. The droplets were subjected to amplification followed by transferring to a Droplet reader (Bio-Rad) and were analyzed with the Quanta Soft analytical software package (Bio-Rad) (14). As shown in Figure 2, the *MYD88* p.L265P mutation was detected in DNA extracted from tumor tissue and ctDNA obtained from the CSF (CSF-ctDNA). In contrast, the p.V217F mutation was not detected either in the tissue or in the CSF-ctDNA, highlighting the specificity of the assay.

## Discussion

Currently, establishing a diagnosis of CNS lymphomas often requires invasive procedures such as craniotomy or stereotactic biopsy. Minimally invasive procedures such as MRI, CSF-cytology, and flow cytometry, have limited value due to low specificity and sensitivity (15, 16). In our case, MRI suggested involvement of the CNS by the patient's known lymphoma. However, CSF-cytology and flow cytometry did not provide conclusive results and an intracranial biopsy was performed to establish a definite diagnosis. CSF cytology and flow cytometry, although specific, require the presence of intact tumor cells in the CSF. As a result, these methodologies have a high rate of false negative results (17–19). In addition, treatment with chemotherapy and steroids may have a negative impact on the ability to detect intact tumor cells in the CSF by microscopic examination, but not on the detection of CSF-ctDNA. Therefore, analysis of CSF-ctDNA is a promising methodology for the diagnosis of CNS lymphoma. Our study demonstrates the potential of CSF-ctDNA analysis for the *MYD88* p.L265P mutation as a promising complementary method to assist in establishing the diagnosis of CNS lymphoma.

*MYD88* mutations in cancers mostly occur as a single base substitution at c.794T > C resulting a leucine to proline (L265P) amino acid change in the Toll/IL-1 receptor domain. This genetic alteration causes recruitment of the *MYD88* protein to the cytoplasmic tail of the TLRs to form an active complex and subsequent activation of the NF-kB signaling pathway (12, 20, 21). The *MYD88* p.L265P mutation is reported 2,103 times in the COSMIC database. Out of 2,103 reports, 2,097 cases are in hematologic malignancies, with rare instances in lung (*n* = 2), peritoneum (*n* = 1), large intestine (*n* = 1), and prostate tumors (*n* = 1) (22). A less common *MYD88* mutation (p.V217F) has been reported in lymphomas (23). Therefore, in our study, we analyzed CSF-ctDNA for the presence of both p.L265P and p.V217F using ddPCR, and our results demonstrate the presence of the p.L265P mutation in both, CNS tissue and CSF-ctDNA. After primary CNS lymphoma, testicular lymphomas (the primary lymphoma of the patient reported here) have the second highest prevalence of *MYD88* mutations (11). To our knowledge, there is only one previously published study, showing detection of the *MYD88* p.L265P mutation in the 9/32 tested CSF from patients with PCNSL and lymphoplasmacytic lymphoma (24).

## Concluding remarks

In summary, our study demonstrates that ddPCR analysis of CSF-ctDNA is a promising methodology to detect *MYD88* mutations in patients with CNS lymphoma. This could potentially be used as an alternative and minimally-invasive method for diagnosis. However, further investigation in a larger cohort of patients is required to establish this assay as a routine clinical test.

## Ethics statement

This project was approved by the institutional review board of the University of Texas Health Science Center at Houston and Memorial Hermann Hospital, Houston, TX. Residual tumor tissue and CSF were analyzed with written consent from the patient. In addition, the written informed consent was obtained from the patient for the publication of this case report.

## Author contributions

LB designed and supervised the study and data analysis. SZ, JW, and DD designed and performed the experiments. GL and J-JZ are responsible for clinical information gathering, sample collection, and patient consent. SZ and YE prepared the manuscript.

### Conflict of interest statement

The authors declare that the research was conducted in the absence of any commercial or financial relationships that could be construed as a potential conflict of interest.
